# A diphtheria toxin resistance marker for *in vitro* and *in vivo* selection of stably transduced human cells

**DOI:** 10.1038/srep14721

**Published:** 2015-09-30

**Authors:** Gabriele Picco, Consalvo Petti, Livio Trusolino, Andrea Bertotti, Enzo Medico

**Affiliations:** 1 Candiolo Cancer Institute—FPO, IRCCS, Candiolo, Torino, Italy; 2 University of Torino, Department of Oncology, SP 142, Km 3.95, 10060 Candiolo, Torino, Italy

## Abstract

We developed a selectable marker rendering human cells resistant to Diphtheria Toxin (DT). The marker (DT^R^) consists of a primary microRNA sequence engineered to downregulate the ubiquitous DPH2 gene, a key enzyme for the biosynthesis of the DT target diphthamide. DT^R^ expression in human cells invariably rendered them resistant to DT *in vitro*, without altering basal cell growth. DT^R^-based selection efficiency and stability were comparable to those of established drug-resistance markers. As mice are insensitive to DT, DT^R^-based selection can be also applied *in vivo*. Direct injection of a GFP-DT^R^ lentiviral vector into human cancer cell-line xenografts and patient-derived tumorgrafts implanted in mice, followed by systemic DT administration, yielded tumors entirely composed of permanently transduced cells and detectable by imaging systems. This approach enabled high-efficiency *in vivo* selection of xenografted human tumor tissues expressing ectopic transgenes, a hitherto unmet need for functional and morphological studies in laboratory animals.

Stable transduction, i.e. permanent integration of exogenous genetic material in the cellular genome, is widely employed in basic and applied biomedical research^1^. In the case of human cells, this is typically achieved by transduction with an expression vector encoding resistance proteins for cytotoxic antibiotics like G418, puromycin, hygromycin and others. Pharmacologic selection then yields a cell population constitutively expressing the resistance marker, plus additional genetic elements of interest. However, current drug/marker systems cannot be used for *in vivo* selection of transduced human cells propagated in animals as xenografts, since the drugs are also lethal for the host animals. This lack of *in vivo* selectable markers is a crucial drawback for patient-derived cancer xenografts (PDXs), which are directly propagated from human tumour tissue into immunocompromised mice and for which no procedures for *in vivo* stable transduction have been found to work efficiently^2^,^3^,^4^,^5^,^6^. We reasoned that, in principle, injection of a viral expression vector into the PDX mass followed by treatment of the mouse with a drug exerting species-specific killing activity only on human non-transduced cells would enable selection of stably transduced PDXs, for marker studies or for proof-of-concept therapeutic target validation. We considered that diphtheria toxin (DT) has the required pharmacological properties: it invariably kills human cells^7^ using the ubiquitously expressed transmembrane heparin-binding EGF-like growth factor (HBEGF) as a receptor^8^, but has no effect on mouse tissues, because the murine Hbegf does not bind DT^9^,^10^. Moreover, DT has been successfully employed for cell lineage ablation in animal models^10^,^11^. A well-documented strategy to induce DT resistance in human cells is the blockade of Diphthamide Biosynthesis Protein 2 (DPH2), either by expression of a dominant-negative protein^12^ or by gene inactivation via insertional mutagenesis^13^. DPH2 catalyzes a key step in diphthamide biosynthesis, a histidine modification process known to occur only on His715 of Eukaryotic Elongation Factor 2 (EEF2)^14^. After HBEGF-mediated DT internalization, DT inhibits EEF2 by catalyzing the transfer of NAD+ to diphthamide^15^. In the absence of active DPH2, His715 is not converted to diphthamide and the cell is insensitive to DT. Additional genes, including DPH5, DPH6, and DPH7, have been shown to encode proteins essential for diphthamide formation and DT-mediated toxicity in human cells^16^,^17^,^18^. Notably, cells lacking diphthamide have no distinct phenotypes except their resistance to DT^19^. Therefore, the interruption of diphthamide biosynthesis represents an attractive strategy to render human cells resistant to DT without major biological consequences.

To develop a selectable marker conferring resistance to DT, we considered an RNA interference approach, using short hairpin sequences inserted into a primary microRNA transcript backbone (shRNAmirs). This design adds a Drosha processing site to the hairpin construct that has been shown to greatly increase knockdown efficiency^20^. Four to six different shRNAmir sequences were tested for each of the key diphthamide biosynthesis genes.

## Results

### DPH2 silencing renders human cells resistant to diphtheria toxin *in vitro*

In principle, to kill human cells, diphtheria toxin requires the presence on the cell surface of its receptor, HBEGF, and of diphthamide biosintesis proteins, in particular of DPH2. We therefore evaluated HBEGF and DPH2 expression in a series of gene expression datasets: (i) 151 CRC cancer cell lines^21^, (ii) 515 CRC PDX (manuscript in preparation), and (iii) expression data for colorectal, glioblastoma, head and neck and pancreatic cancer obtained from The Cancer Genome Atlas via the CBioPortal^22^. As reported in Supplementary Fig. 1, both HBEGF and DPH2 are well expressed across all datasets.

**Figure 1:**
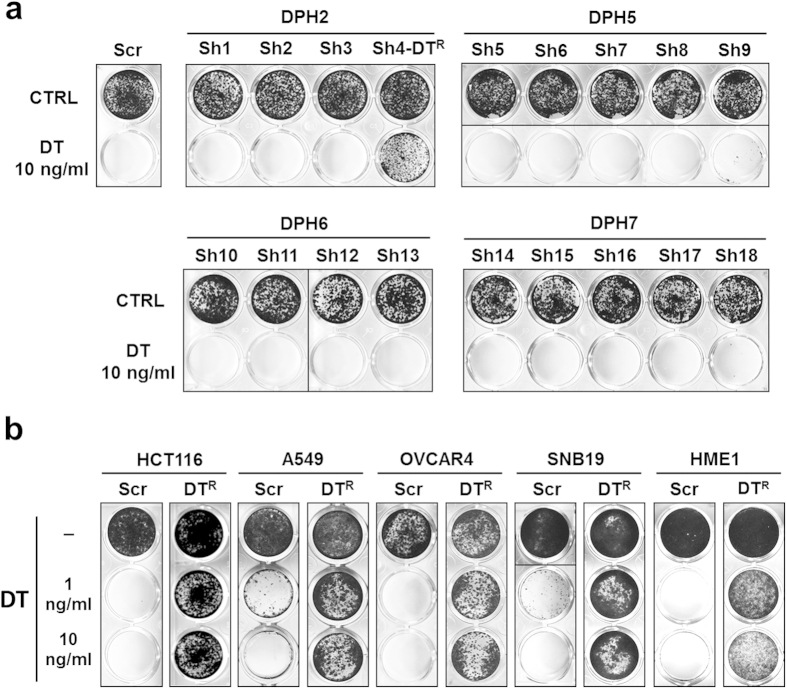
DPH2 silencing renders human cell lines resistant to DT. (**a**) Crystal violet staining of HCT116 cells transduced with Scramble or shRNAmirs targeting DPH2 (sh1-4), DPH5 (sh5-9), DPH6 (sh10-13) and DPH7 (sh14-18), grown in the absence or presence of DT (10 ng/ml) for one week. The only construct conferring resistance to DT was sh4, hence renamed DT^R^. (**b**) Crystal violet staining of human normal (HME1) and neoplastic cell lines (HCT116, colon; A549, lung; OVCAR4, ovary; SNB19, glioblastoma) transduced with Scramble or DT^R^ vector and grown in the absence or presence of DT (1 ng/ml or 10 ng/ml) for one week.

To validate DT as a selective agent, we verified its activity on a panel of cell lines of different tissue origin, expressing variable levels of HBEGF^23^. DT activity was indeed always high and independent of HBEGF expression levels (Supplementary Fig. 2). To assess if silencing of the genes involved in diphthamide biosynthesis confers resistance to DT, HCT116 colorectal cancer (CRC) cells, expressing intermediate levels of HBEGF, were chosen as a model and transduced with 18 different shRNAmir constructs, targeting the *DPH2, DPH5, DPH6* and *DPH7* transcripts (Supplementary Table 1). Transduced cells were then tested for their response to DT (Fig. 1a) and for downregulation of the target transcript (Supplementary Table 2). Among all the tested shRNAmirs, only the construct #4, targeting DPH2, was found to induce both robust downregulation of DPH2 mRNA levels and strong resistance to DT. This shRNAmir sequence is hereafter referred to as DT^R^ (“diphtheria toxin resistance”). Efficient downregulation of DPH2 and induction of resistance to DT was confirmed in three additional human cancer cell lines derived from different tissues and in a non-transformed human breast epithelial cell line (Fig. 1b, Supplementary Table 2). Notably, cells expressing the DT^R^ marker were not impaired in their growth rate (Supplementary Fig. 3). To assess if DT^R^ can be efficiently employed as a selectable marker *in vitro*, we exploited the expression cassette for GFP and puromycin resistance included in the lentiviral DT^R^ vector. HCT116 were transduced with DT^R^ at low MOI to obtain around 2% of GFP-positive (GFP+) cells, and then selected in the presence of either DT (10 ng/ml) or puromycin (2 ng/ml) for two weeks. Both selections were found to strongly increase the GFP+ fraction (>95%; Supplementary Fig. 3). To verify the stability of the resistant phenotype, both selected populations were grown in the absence of DT or puromycin for one month, and found to retain a very high fraction of GFP+ cells (~80% and ~90% for puromycin and DT-selected cells, respectively; Supplementary Fig. 4). Persistence of the high GFP+ fraction was also observed after multiple freeze-thaw cycles (not shown).

### Human cells transduced with DT^R^ become resistant to diphtheria toxin *in vivo*

**Figure 2:**
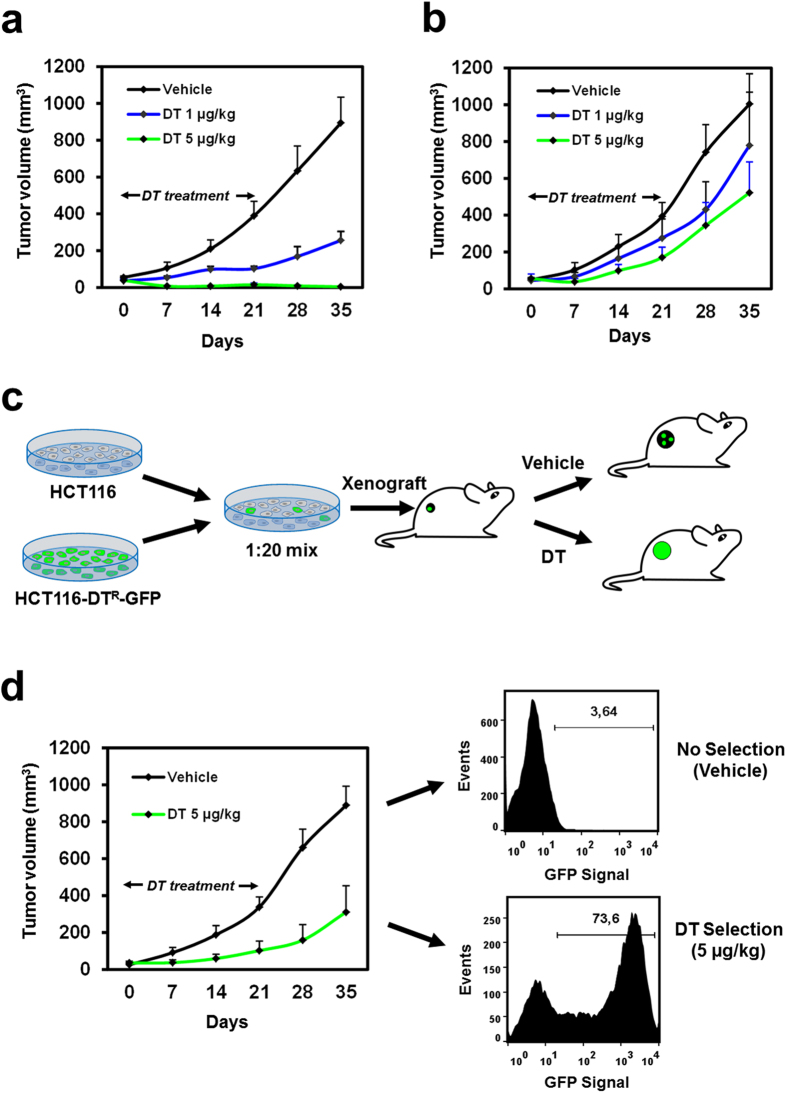
DT^R^ transduced cells are resistant to DT *in vivo.* (**a**,**b**) Tumor growth curves of xenografts from HCT116 cells transduced with either the Scramble vector (**a**) or the DT^R^ vector (**b**) in nude mice treated with DT (1 μg/kg or 5 μg/kg) or vehicle (n = 5). (**c**) Schema of the *in vivo* selection experiment. Nude mice xenografts obtained from a mixture of DT^R^-transduced (GFP-positive) and parental (GFP-negative) HCT116 cells (1:20 ratio) were treated with DT (5 μg/kg) or vehicle for three weeks. It is expected that xenografts grown in the presence of DT are enriched in GFP-positive cells. (**d**) Tumor growth curves (n = 4) in the course of the DT selection process, followed by flow-cytometry analysis of GFP levels in representative tumors explanted from unselected (up) and selected (bottom) cohorts, as indicated.

To assess if DT^R^-transduced cells maintain DT resistance also in the context of a living organism, HCT116 cells transduced with DT^R^ or with a control vector were grown as subcutaneous xenografts in nude mice. When xenografts reached ~50 mm^3^, mice were treated with DT or with vehicle for three weeks. As shown in Fig. 2a, treatment of control HCT116 xenografts with 1 μg/kg DT strongly reduced their growth rate, and 5 μg/kg DT induced complete tumor regression, which persisted also after DT suspension. Conversely, DT^R^ transduced cells resisted to both doses of DT and continued growing in the presence of DT and after its withdrawal (Fig. 2b). Notably, in the absence of DT, DT^R^-transduced HCT116 xenografts displayed a growth rate similar to that of control xenografts (Fig. 2a,b, “Vehicle” lines), confirming that DT^R^ expression has no major effects on cancer cell growth. As previously reported, no adverse effects were observed in mice treated with DT^10^,^24^.

To verify the possibility of using DT^R^ as a marker for *in vivo* selection of transduced cells, we designed the experiment illustrated in Fig. 2c. Briefly, GFP-DT^R^-transduced and control HCT116 cells were mixed in a 1:20 ratio, to obtain a heterogeneous population in which the DT^R^-expressing, GFP+ fraction was around 5%. The mixed cell population was then implanted and grown in nude mice xenografts in the absence or presence of DT for three weeks, followed by two additional weeks without treatment. As shown in Fig. 2d, treatment with 5 μg/kg DT did not induce tumor regression but only a reduced growth rate. After two weeks from the end of the treatment, flow cytometry on the explanted tumors revealed a striking enrichment of GFP+ cells in the DT-treated arm (Fig. 2d). Altogether, these results show that *DPH2* silencing by DT^R^ confers resistance to DT also *in vivo* and can be exploited for *in vivo* selection of transduced cells.

### *In vivo* transduction and selection for DT^R^ expression in xenografts from human cell lines and tumors

**Figure 3:**
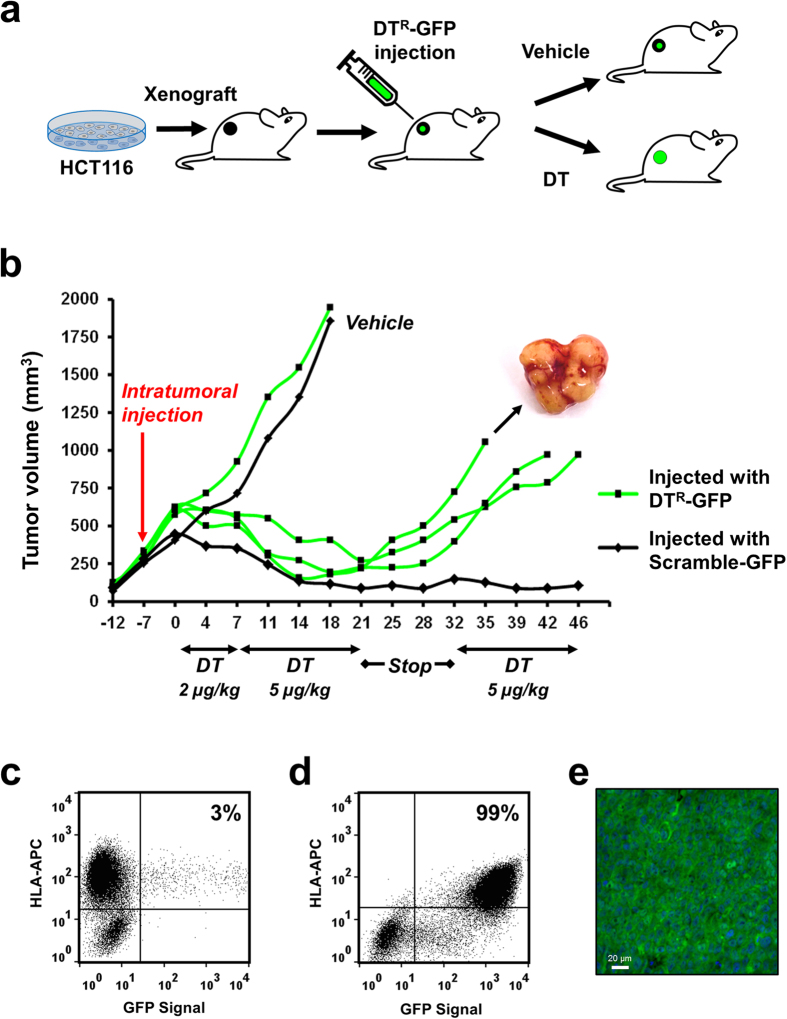
*In vivo* selection of DT transduced tumors. (**a**) Schema of the *in vivo* DT^R^ transduction and selection experiment. (**b**) Growth curves of HCT116 xenografts in CD1-nude mice transduced *in vivo* by intratumoral injection of scramble-GFP or DT^R^-GFP concentrated lentiviral particles. A week after vector injection, DT was administered at increasing concentrations for three weeks, followed by 12 days of drug withdrawal and two additional weeks of treatment. One control tumor for each vector was allowed to grow in the absence of DT as a control. (**c**,**d**) Flow cytometry analysis of the fraction of GFP+ cells in unselected (**c**) or DT-selected (**d**) tumors. (**e**) Fluorescence micrograph displaying GFP expression in a representative DT^R^-injected, DT-selected HCT116 xenograft subsequently re-implanted and grown in the absence of DT.

Generation of genetically modified xenografts is easily accomplishable with most neoplastic cell lines by *in vitro* transduction and selection, followed by implant in mice. In the case of PDXs however, such procedure is typically not applicable or poorly efficient. We therefore sought to verify if direct intratumoral injection of the DT^R^ vector in xenografts from human cell lines and patient-derived tumors, followed by DT treatment of mice, could lead to the development of xenografts significantly enriched in transduced cells. To this aim, HCT116 xenografts in nude mice were directly transduced by intratumoral injection of concentrated lentiviral particles of GFP-DT^R^ or GFP-control (scramble) vector. After one week, cell suspensions were obtained from a first set of transduced tumors for flow cytometry analysis, which detected a fraction of GFP+ cells around 1% for both vector types (average of 5 measurements = 0.97% +/− 1.70%). In a parallel set of xenografts, one week after transduction, mice were treated with DT for three weeks, followed by two weeks of suspension, after which DT was maintained until tumor explant (Fig. 3a). While all tumors displayed marked shrinkage after three weeks of DT treatment, only DT^R^-transduced tumors resumed growth after the initial shrinkage. The regrown tumors featured a multinodular mass, suggestive of parallel growth of multiple resistant subclones from the areas of vector injection (Fig. 3b, photo insert). GFP-DT^R^-transduced and selected xenografts revealed a striking enrichment in human GFP+ cells with respect to transduced tumors grown in the absence of selection (99% vs 3%; Fig. 3b,c).

Intriguingly, the high fraction of transduced cells and the DT resistance were stably maintained over multiple passages in mice, also in the absence of DT selective pressure (Supplementary Fig. 5). Moreover, growth of GFP-DT^R^-transduced tumors implanted subcutaneously or in the peritoneum cavity could be longitudinally followed by measuring *in vivo* GFP fluorescence signal as a surrogate value of the tumor mass volume (Supplementary Fig. 6).

**Figure 4:**
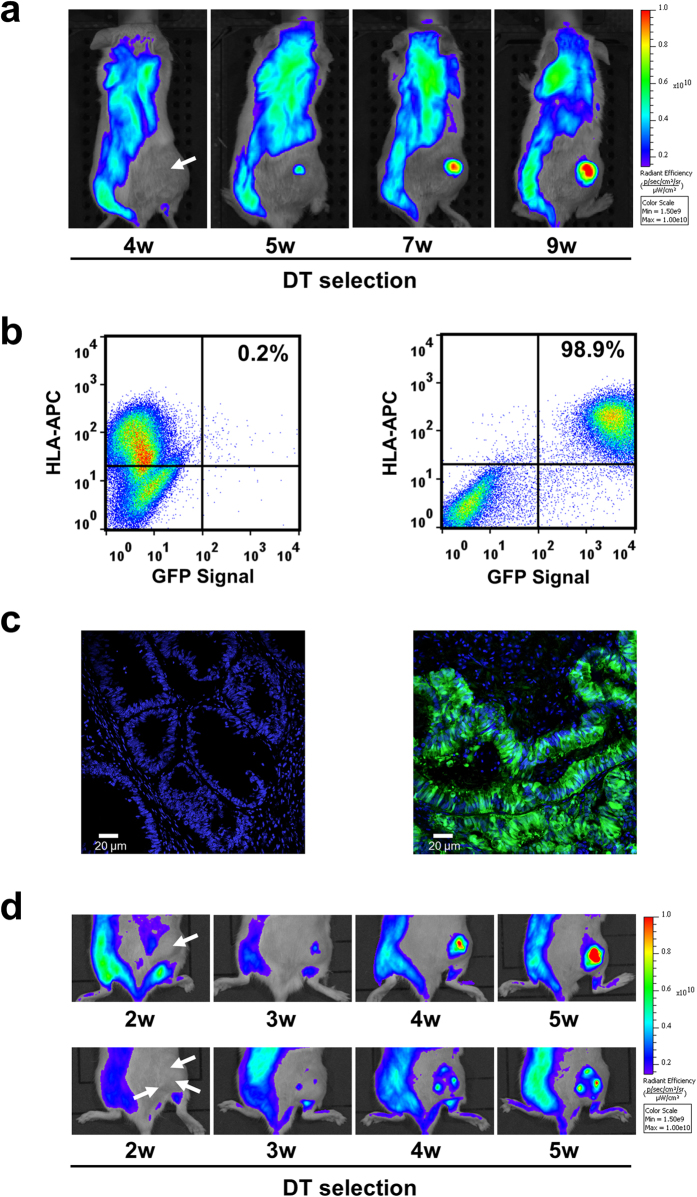
*In vivo* DT^R^ transduction and selection of CRC patient-derived xenografts. (**a**) *In vivo* selection of a PDX grown in a NOD-SCID mouse and directly injected (white arrow) with the DT^R^-GFP lentiviral vector, imaged by IVIS. After about five weeks of selection, a GFP-positive area emerges in the tumor mass. The xenograft area was shaved to reduce fur-derived background. (**b**) Flow cytometry analysis for GFP and human HLA-APC signals of unselected (left) or DT-selected PDXs (right), explanted at the end of the selection period. (**c**) Fluorescence micrograph displaying GFP expression in unselected (left) and DT^R^-injected, DT-selected PDX subsequently re-implanted and grown in the absence of DT (right). (**d**) IVIS imaging of an additional CRC PDX model *in vivo* injected with DT^R^-GFP lentiviral particles in a single or multiple site (upper and lower panels, respectively) and selected with DT for six weeks. In this model GFP-positive regions became already detectable after three weeks of selection.

As a proof of concept of the efficiency of the DT^R^ vector system in an additional preclinical platform, we reproduced the same experimental procedure employing human CRC PDXs instead of cell line xenografts. A preliminary test confirmed that also these PDX tumors were sensitive to DT, independently of their genetic makeup (Supplementary Fig. 7). One CRC PDX model was transduced *in vivo* by a single intratumoral injection of GFP-DT^R^ lentiviral particles. Also in this case transduction efficiency was estimated as above to be around 1%. The DT selection process was then monitored by *in vivo* tracking of the GFP signal in the transduced tumors. To reduce the background due to the white fur, the skin surrounding the xenograft was shaved. After about five weeks of selection, the GFP signal became detectable in the tumor mass, and kept increasing in the following weeks (Fig. 4a). As confirmed by flow cytometry and microscopic analysis, almost all human cancer cells were positive for GFP (Fig. 4b,c). Also in this case lentiviral transduction of PDX tumors with GFP-DT^R^ did not alter tumor morphology and proliferative index (Ki67 expression; Supplementary Fig. 8) or the expression of differentiation and functional markers commonly employed for CRC classification (CDK20, CDX2 and b-catenin; Supplementary Fig. 9. In addition, we found that the fraction of GFP positive cells remains very high (92%) after multiple passages of propagation of the transduced PDX in the absence of DT selective pressure (Supplementary Fig. 10).

To further explore the possible impact of DT^R^ transduction and DT selection, we performed global mRNA expression profiling of: (i) one CRC PDX before any treatment; (ii) its derivative obtained after *in vivo* DT^R^ transduction and DT selection (“P0”); (iii) three additional PDX derivatives propagated from the first DT-selected PDX at different passages (“P1”, “P2” and “P4”), in the absence of DT. In accordance with the high GFP+ fraction observed by flow cytometry, downregulation of the DPH2 transcript was maintained in all DT^R^ samples respect to the parental PDX, even at four passages after transduction and selection (Supplementary Fig. 11a). Correlation analysis revealed that the range of correlations between the parental PDX and its DT^R^ derivatives was similar to that of correlations between the various DT^R^ derivatives (Supplementary Fig. 11b). Interestingly, hierarchical clustering of all samples (PDX and DT^R^ derivatives) based on the global expression profile revealed that P1-P4 DT^R^ samples are even closer to the parental PDX than the P0 DT^R^ sample, that probably underwent transient modulation of gene expression following cell death and tissue reorganization induced by DT selection (Supplementary Fig. 11c). Overall, these results show that no major changes in the global transcriptional profile occurred in the CRC PDX after DT^R^ transduction and DT selection.

Efficiency of the DT^R^ selection procedure was confirmed in a second PDX model, transduced with single or multiple injections of GFP-DT^R^ lentiviral particles. During DT selection, single or multiple GFP+ areas became detectable, reflecting growth of GFP+ cells in proximity of the injection sites (Fig. 4d). Also in this case, the selection process therefore led to the formation of a growing GFP+ tumor.

## Discussion

In this work we demonstrate that an artificial shRNAmir sequence targeting the DPH2 transcript invariably confers resistance to DT in human cells, and that this sequence can be effectively exploited to select permanently transduced human cells both *in vitro* and *in vivo*. A major limitation of currently available selectable markers is the impossibility to apply any selection process *in vivo*: the drugs used for selection are toxic for all eukaryotic cells and therefore would be lethal if administered to a mouse hosting human cells, e.g. a human tumor xenograft. This restriction is not particularly relevant for cell lines, which can be transduced and selected *in vitro* and then implanted or injected into immunocompromised mice. It is instead critical for PDXs, for which *in vivo* viral-mediated transduction has only been successful with constructs that directly provide a selective advantage during outgrowth^25^. Such methodology is not suitable for basic tumor marking or for therapeutic proof-of-concept studies in which expression of the sequence of interest would be detrimental to cancer cells. *Ex vivo* transduction strategies, by which cells are separated from explanted tissues, transduced and reimplanted, have also been described^25^,^26^. However, the complexity and low efficiency of this approach pose severe limits to their practical application. Moreover, *in vitro* culture of PDX-derived cancer cells has been shown to deeply and irreversibly alter their gene expression profile, and therefore their functional state^27^. In addition to the possibility of *in vivo* selection, other interesting features characterize DT^R^ as a marker. Typically, a resistance marker encodes for a protein capable of rendering transduced cells resistant to a toxic compound. The coding sequence for such marker is several hundred nucleotides long, and requires a dedicated promoter or an internal ribosome entry site for efficient translation, which substantially increases vector size. This in turn reduces the size of additional genetic elements that can be efficiently cloned and transduced^28^. In this respect, the DT^R^ sequence has two major advantages: (i) it is only 320 nucleotides long; (ii) it can be directly inserted between the promoter and the coding sequence of interest, or after its stop codon, before the polyadenylation site. In this way, the nascent transcript is processed to yield both the mRNA of interest and the DT^R^ marker, without the need for additional promoters or internal ribosome entry sites. Our results show that this is the case: with DT^R^ inserted downstream from GFP, all DT-selected cells displayed strong GFP fluorescence. The high efficiency of DT^R^ is also particularly relevant to tumor heterogeneity, which should be preserved as much as possible after DT^R^ transduction and DT selection. We noticed that intratumoral injection of high titer DT^R^ lentiviral particles in a PDX generates about 0.2–1% of stably transduced cells. Considering that the estimated number of cancer cells in a tumor is about 10^8^ per cubic centimeter^29^, we therefore estimate that a typical *in vivo* transduction of a 200 mm^3^ PDX generates about 1–2 × 10^5^ stably transduced cells, which should allow preserving tumor heterogeneity.

In principle, abrogation of diphthamide biosynthesis could affect the function of the involved protein, EEF2, and consequently influence protein translation. However, it is unlikely that interference with the diphthamide biosynthetic pathway provokes relevant biological alterations, given that tumor morphology and growth rate were not modified in our experiments, in accordance with previous findings^19^. Moreover, the fraction of GFP positive cells remains very high after multiple passages of propagation in the absence of DT selective pressure. This confirms that stable expression of DT^R^ does not impair cell growth even under the stressful conditions of tumor explant and re-implant. In any case, we cannot exclude that off-target activity of DT^R^ could reduce stability or translation of additional mRNAs in transduced cells, albeit with no overt consequences on cellular growth, morphology or gene expression profile. In view of these considerations, how DPH2 gene silencing and *in vivo* DT selection could impact on maintenance of the biological features and heterogeneity of the tumor should be accurately evaluated in each specific experimental setting. Regarding e.g. the employment of DT^R^-transduced PDX to follow drug response *in vivo*, possible interactions between DPH2 silencing and drug efficacy must be carefully assessed, in particular for drugs affecting protein synthesis.

A straightforward way to render human cells resistant to DT would be to abrogate expression of HBEGF, encoding the transmembrane DT receptor. However, due to its mitogenic activity^30^ and to its release as a soluble form after membrane shedding^31^, its silencing is likely to induce phenotypic changes in both transduced and surrounding cells. Similarly, other enzymes required for Dhipthamide formation, like DPH1 and DPH3, are not suitable candidates because they have been involved in the regulation of cell growth and development^32^,^33^. Among the remaining candidate targets, DPH2 was not the only one tested in our screening for DT resistance markers: at least four independent shRNAmirs were tested against each of the *DPH5, DPH6* and *DPH7* genes. None of them was capable of inducing detectable DT resistance. Indeed, the extent of target mRNA downregulation was quite limited for most of them. These results imply that, in principle, alternative and more efficient shRNAmirs targeting the same genes could promote DT resistance, or otherwise that downregulation of *DPH5, DPH6* and *DPH7* brings a selective disadvantage to transduced cells.

*In vivo* stable transduction with GFP/luciferase based vectors enabled by DT^R^ is expected to significantly improve the spectrum and performance of tumor-tracking applications. This is particularly true for intraperitoneal and other orthotopic models, where caliper measurements are not suitable, and fluorescence or bioluminescence imaging represent a cost effective, simple approach. In the case of GFP, used in the present work, absorption and/or autofluorescence by animal tissues make monitoring of labeled cells in deep locations problematic^34^. Notwithstanding these limitations, the growth of GFP-DT^R^ transduced tumors was effectively monitored by *in vivo* imaging, even when the xenografts were intraperitoneal. This indicates that the DT^R^ selection system enables robust expression of the co-selected coding sequences, as confirmed by the high levels of GFP signal detected by flow cytometry and fluorescence microscopy in selected cells and xenografts. Moreover, stable DT^R^ expression allows rapid DT re-selection of transduced cells, should their fraction become lower.

Additional possible applications of the DT^R^ module include transduction of PDXs with therapeutically relevant sequences, like cDNAs for proteins modulating drug response, or short RNA hairpins interfering with gene expression of undruggable or poorly actionable oncogenic drivers. To this aim, development of efficient multi-module expression vectors will be greatly facilitated by the small size and pri-miRNA nature of DT^R^. This will however require careful assessment of possible interferences between post-transcriptional processing of DT^R^ and other modules, or between these and viral vector assembly^35^. In light of the rapidly increasing availability and use of PDX models for preclinical experimentation, the DT^R^ system presented here offers an unprecedented opportunity for direct genetic manipulation of human tumor xenografts.

## Methods

### Cell culture and reagents

All cell lines were purchased from American Type Culture Collection (ATCC). Cell lines were maintained in culture following supplier guidelines. Cells were ordinarily supplemented with FBS at different concentrations, 2 mM L-glutamine, antibiotics (100 U/mL penicillin and 100 mg/mL streptomycin) and grown in a 37 °C and 5% CO2 air incubator. In particular, HCT-116 and A549 cell line were maintained in RPMI-1640 medium, supplemented with 10% FBS. Hutu-80 and HeLa cells were maintained in DMEM, supplemented with 10% FBS. HME1 cells were cultured DMEM/F-12 supplemented with 20 ng/mL EGF, 10 μg/mL insulin, and 100 μg/mL hydrocortisone and 10% FBS. Proliferation assays were performed seeding 5–15 × 10^3^ cells in 24-well plates or 5 × 10^4^ cells in 6-well plate. After 24 hours, medium was replaced adding DT (D0564, Sigma) reconstituted to 0.5 mL with sterile distilled water. After one week of treatments, cells were fixed with a solution of 3% Paraformaldehyde plus 1% glucose and then stained with 0.05% Crystal Violet in Distilled Water.

### RNA interference

For stable silencing of DPH2 and DPH6 genes, pGIPZ Gene Sets (RHS4531-NM_001384 and RHS4430-NM_080650) and pGIPZ-GFP-scrambled-shRNAmir lentiviral vectors were purchased from Open Biosystems. For inducible silencing of DPH7 and DPH5, pTRIPZ shRNA Gene Sets (RHS4740-NM_138778 and RHS4740-NM_001077395) plus pTRIPZ-scrambled-shRNAmir lentiviral vectors were purchased from Open Biosystems. Details of individual clones are reported in Supplementary Table 2.

The Open Biosystems protocols were followed for bacterial culture growth and for recombination checks. pGIPZ and pTRIPZ plasmids were purified with Miniprep or Maxiprep kits (Qiagen) and DNA concentration was measured by Nanodrop 1000. All lentiviral constructs were transfected into HEK293T cells plated in a 6-well plate at a density of 5 × 10^4^ cells per well one day prior to transfection, using Lipofectamine 2000 (Invitrogene). Supernatants were harvested after 48 hours, filtered through a 0.45-μm filter, and used to infect cells in the presence of 4 μg/mL of polybrene. Cells were selected in 2 μg/mL puromycin or directly in diphtheria toxin at the indicated doses. For cells transduced with inducible pTRIPZ vector, shRNAmir expression was induced adding doxycycline (1 μg/mL) in to the growth medium and RFP fluorescence was assessed by FACS analysis before performing growth assay and Q-RT-PCR. For *in vivo* injection, lentiviral particles obtained from the supernatants of 7 15-cm plates of HEK293 cells transfected as previously described^36^, were concentrated by ultracentrifugation. Concentration of the viral p24 antigen was determined by HIV-1 p24 core profile ELISA (PerkinElmer Life Sciences).

### Q-RT-PCR and gene expression profiling

Total RNA was extracted using the miRNeasy Mini Kit (Qiagen), according to the manufacturer’s protocol. The quantification and quality analysis of RNA was performed by Thermo Scientific Nanodrop 1000 and Bioanalyzer 2100 (Agilent). DNA was transcribed using iScript RT Super Mix (BioRad) following the manufacturer’s instructions. Q-RT-PCR was performed in triplicate on ABI PRISM 7900HT thermal cycler (Life Technologies) with SYBR green dye. The sequences of the primers (Sigma Aldrich) used for gene expression analyses of DPH2, DPH5, DPH6 and DPH7 genes are reported in Supplementary Table 3. For gene expression profiling, synthesis of cDNA and biotinylated cRNA was performed using the IlluminaTotalPrep RNA Amplification Kit (Ambion), according to the manufacturer’s protocol using 500 ng of total RNA. Quality assessment and quantification of cRNAs were performed with Agilent RNA kits on Bioanalyzer 2100. Hybridization of cRNAs (750 ng) was carried out using Illumina Human 48 k gene chips (Human HT-12 V4 BeadChip). Array washing was performed using Illumina High Temp Wash Buffer for 10′ at 55 °C, followed by staining using streptavidin-Cy3 dyes (Amersham Biosciences). Probe intensity data were obtained and normalized using the Illumina Genome Studio software (Genome Studio V2011.1) and further processed with Excel software. Correlation analysis was based on calculation of the Pearson correlation coefficient using all probe signals. Hierarchical clustering of PDX samples using euclidean distance and average linkage was performed using the GEDAS software^37^.

### Flow cytometry

GFP expression analysis of *in vitro* cultured cells was performed by flow cytometry: cells were trypsinized, diluted in a 1% paraformalhdeide-2% FBS solution, stained with DAPI (D9542, Sigma) and analyzed with FACS flow cytometer (*CyAn*™, DAKO).

Explanted tumors were dissected by bistoury and resuspended in collagenase (C5138, Sigma), Trypsin and Medium 199 (Sigma), for 30 minutes at 37 °C. After inhibition of tripsine and centrifugation, pellets were washed with HBSS 5% FBS and resuspended in red blood cell lysis buffer (NH_4_Cl (155 nM), KHCO3 (10 mM), 0,1 mM EDTA) for 3 minutes. After incubation with DNAse (5 ug/ml) for 5 minutes at 37 °C, cells were counted and 10 × 10^6^ cells were stained with human HLA-ABC antibody (555555, BD Pharmingen) for 45 min in ice. Subsequently, for flow cytometry analyses, cells were filtered with 40 μm filters and stained with DAPI.

### Animal models and live imaging

All animal procedures and care administered were approved by the Ethical Commitee of the University of Turin and the Italian Ministry of Health. The methods were carried out in accordance with the approved guidelines. Nonobese diabetic/severe combined immunodeficient (NOD/SCID) mice and CD-1 nude female mice were purchased from Charles River Laboratories (Calco, Italy) and maintained in hyperventilated cages. HCT116 xenografts were established by subcutaneous inoculation of 2 × 10^6^ cells into the right posterior flank of 5–6 week-old mice. Tumor size was evaluated by caliper measurements and the approximate volume of the mass was calculated using the formula (d/2)^2^ × D/2, where d is the minor tumor axis and D is the major tumor axis. HCT116 secondary grafts, employed for *in vivo* transduction, were obtained starting from material explanted from HCT116 primary xenograft: tumors were cut into 25 to 30 mm^3^ pieces and re-implanted in the flank of the mice. Each intratumoral injection was performed with approximately 1 mg of lentiviral particles diluted in physiological saline solution. Tumor cells were injected subcutaneously into immunodeficient mice as described above (2 × 10^6^ cells/mouse). After 2 weeks, mice bearing tumors of approximately 100 mm^3^ were selected and were randomly divided into 3 groups of 6 mice each. *In vivo* transduction experiments were conducted injecting vectors expressing the shRNAmir sequences and GFP reporter directly into the tumor mass. Single or multiple intratumoral injection of 1 mg of lentiviral particles were performed by a 0.5 ml syringe. The same procedure was employed to transduce PDXs, in which injection of lentiviral particles was performed when tumors reached approximately 200 mm^3^. For selection of cell xenografts, DT was administered three times a week at the indicated dosages and schedules. For PDX selection, DT was administered three times a week (5 μg/kg) until tumor explant. *In vivo* quantitative and qualitative analysis of GFP expression was performed by using the IVIS® Lumina II imaging and the Living Image software version 3.0 (Caliper Life Science). The GFP filter sets in the system include emission filter (515–575 nm) and excitation filter (445–490 nm). Images of the mice were generated by setting, respectively, 1.5E9 and 1E10 as minimum and maximum values in the color scale. The ROI measurements were individually marked as regions of interest (ROI) in the Living Image software, setting auto ROI detection with 20 as auto detection threshold.

### Immunohistochemistry and confocal microscopy

To detect GFP expression, explanted tumors were fixed by immersion in formalin for 24 hours at 4 °C. Subsequently, the specimens were treated with 30% sucrose solution to protect them from cryopreservation, then embedded in Optimal Cutting Temperature (OCT) compound (Bio-Optica) and stored at −80 °C until sectioning. Subsequently, 12 μm thick sections were cut with a cryomicrotome (Leica CM 3050 S) and collected in Superfrost Plus Slides (Thermo Scientific). Before staining, microscope slides were treated with PBS to remove OCT and stained with DAPI (Sigma). The fluorescent light emitted by Turbo-GFP and DAPI was evaluated on a Leica TCS SP2 AOBS confocal microscope. Images were generated with the LAS AF Leica Application Suite software (Leica) at 20X of magnification. For IHC, formalin-fixed, paraffin-embedded tissues explanted from PDX were partially sectioned (4 μm thick) using a microtome. 4-μm paraffin tissue sections were dried in a 37 °C oven overnight. Slides were deparaffinized in xylene and rehydrated through graded alcohol to water. Endogenous peroxidase was blocked in 3% hydrogen peroxide for 30 minutes. Microwave antigen retrieval was carried out using a microwave oven (750 W for 10 minutes) in 10 mmol/L citrate buffer, pH 6.0. Slides were incubated with monoclonal mouse anti-human Ki67 (clone MIB-1; DAKO), rabbit monoclonal Anti-Cytokeratin 20 antibody [EPR1622Y] (ab76126), mouse Anti-β-Catenin Clone 14/Beta-Catenin (BD Transduction Laboratories) (Catalog No. 610154) and rabbit monoclonal Anti-CDX2 antibody [EPR2764Y] (ab76541) overnight at 4 °C inside a moist chamber. Immunohistochemically stained slides, were scanned with a 40× objective.

## Additional Information

**How to cite this article**: Picco, G. *et al.* A diphtheria toxin resistance marker for *in vitro* and *in vivo* selection of stably transduced human cells. *Sci. Rep.* **5**, 14721; doi: 10.1038/srep14721 (2015).

## Supplementary Material

Supplementary Information
