# Supplementary material for: A diphtheria toxin resistance marker for in vitro and in vivo selection of stably transduced human cells
Source: Sci Rep. 2015 Sep 30;5:14721. doi: 10.1038/srep14721 (PMC4588510; doi:10.1038/srep14721)
Supplement: Supplementary Information [file srep14721-s1.pdf]

A diphtheria toxin resistance marker for *in vitro* and *in vivo* selection of stably transduced human cells

Gabriele Picco, Consalvo Petti, Livio Trusolino, Andrea Bertotti and Enzo Medico

SUPPLEMENTARY INFORMATION

Picco et al., Supplementary Table 1. Details of the shRNA constructs employed in the screening. For each shRNA, columns reports the targeted gene, the lentiviral vector system employed and the mature siRNA antisense sequence. The DT^R construct (Sh4) is highlighted in bold.

Clone ID	Gene	ID	Vector	Mature antisense sequence
V2LHS_265925	DPH2	Sh1	pGIPZ	n/a
V3LHS_367625	DPH2	Sh2	pGIPZ	AGCTGGTCAGGGAACTGCA
V3LHS_367626	DPH2	Sh3	pGIPZ	TTCCCACAGCTCTGACTCA
**V3LHS_382955**	**DPH2**	**Sh4 (DT^R)**	**pGIPZ**	**AGCACAAAGACATCCACCT**
V2THS_134528	DPH5	Sh5	pTRIPZ	TGGTTTACACTCATATACC
V3THS_318426	DPH5	Sh6	pTRIPZ	TTGATTTTGAACAATCTCC
V3THS_318423	DPH5	Sh7	pTRIPZ	TGAACAATCTCCAGAAGCT
V3THS_346448	DPH5	Sh8	pTRIPZ	AACTGGTTCTTCTCCTCGT
V3THS_318422	DPH5	Sh9	pTRIPZ	TGATTTTGAACAATCTCCA
V3LHS_319554	DPH6	Sh10	pGIPZ	TGTCTTGTATCCAAGCTCC
V3LHS_319556	DPH6	Sh11	pGIPZ	TAGATTTGCTAAAGCAACG
V3LHS_319557	DPH6	Sh12	pGIPZ	AGATTTGCTAAAGCAACGA
V3LHS_319558	DPH6	Sh13	pGIPZ	GTCTTGTATCCAAGCTCCT
V2THS_212240	DPH7	Sh14	pTRIPZ	TTTAAGAGAAGTCAACAGC
V3THS_399888	DPH7	Sh15	pTRIPZ	TTAAGAGAAGTCAACAGCT
V3THS_399891	DPH7	Sh16	pTRIPZ	TAAGAGAAGTCAACAGCTT
V3THS_399890	DPH7	Sh17	pTRIPZ	TAAACAAGTATGAAAGGCT
V3THS_301465	DPH7	Sh18	pTRIPZ	AATTGAAAGCAGCAATCCA

Picco et al., Supplementary Table 2. Extent of target transcripts downregulation achieved in cell lines after transduction with lentiviral shRNAmir constructs, analyzed by quantitative real-time PCR. For each cell line, the Table reports the target gene, the clone ID of the lentiviral vector employed, the fraction of residual mRNA and the standard deviation values. Data obtained with the DT^R construct (Sh4) are highlighted in bold. Indeed, only few shMIRs were found to decrease the target transcripts of more than 50%: three for DPH2 and DPH6, one for DPH5 and none for DPH7. The bottom four lines report the silencing activity of DT^R in additional cell lines.

Cell line	Gene Symbol	Clone ID	% of residual transcript	Standard Deviation
HCT116	DPH2	Sh1	39%	5%
HCT116	DPH2	Sh2	129%	12%
HCT116	DPH2	Sh3	46%	6%
**HCT116**	**DPH2**	**Sh4 (DT^R)**	**14%**	**2%**
HCT116	DPH5	Sh5	131%	5%
HCT116	DPH5	Sh6	135%	3%
HCT116	DPH5	Sh7	47%	2%
HCT116	DPH5	Sh8	196%	28%
HCT116	DPH5	Sh9	87%	4%
HCT116	DPH6	Sh10	40%	5%
HCT116	DPH6	Sh11	80%	7%
HCT116	DPH6	Sh12	27%	4%
HCT116	DPH6	Sh13	47%	28%
HCT116	DPH7	Sh14	110%	14%
HCT116	DPH7	Sh15	123%	14%
HCT116	DPH7	Sh16	137%	13%
HCT116	DPH7	Sh17	136%	13%
HCT116	DPH7	Sh18	110%	6%
**A549**	**DPH2**	**DT^R**	**10%**	**1%**
**OVCAR4**	**DPH2**	**DT^R**	**7%**	**1%**
**SNB19**	**DPH2**	**DT^R**	**5%**	**0%**
**HME1**	**DPH2**	**DT^R**	**6%**	**1%**

Picco et al., Supplementary Table 3. List of primers employed for gene expression analysis by real-time PCR. FW: forward primer. RV: reverse primer.

Gene	Sequence FW 5'	Sequence RV 5'
DPH2	CGTGCTTCGTCAACGTTCTG	TGGGTTCTGGGCCTCAAA
DPH5	CCAGAAAGCTTCTTTGACAAAGTG	GATTTTCCAAAGACTGCTCCTTTACT
DPH6	GATCCTGATAAGCATCTTGGGAAA	CTCCACCTTCTCCACAAACATG
DPH7	CCTCTTGGGCTTGGCAGAT	CAGGGCAAGGCTGGACAAT
PGK	AGCTGCTGGGTCTGTCATCCT	TGGCTCGGCTTTAACCTTGT

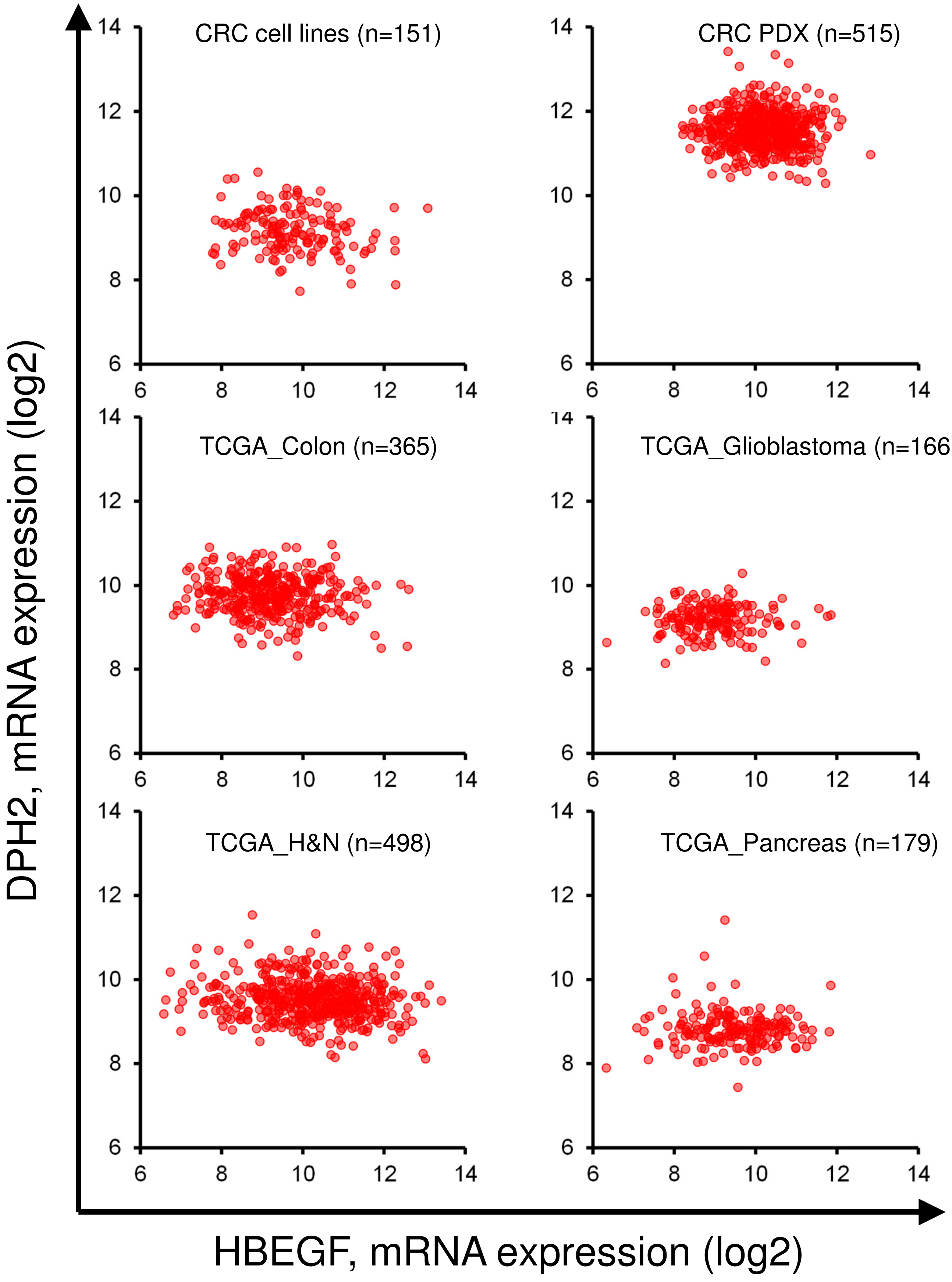

Picco et al., Supplementary Figure 1. HBEGF and DPH2 are well expressed in CRC cell lines, PDXs and tumors, and in other tumor types. Dot plots displaying for each sample, on the x-axis expression of HBEGF, and on the y-axis expression of DPH2. Each dot plot is annotated for the dataset from which expression levels have been obtained.

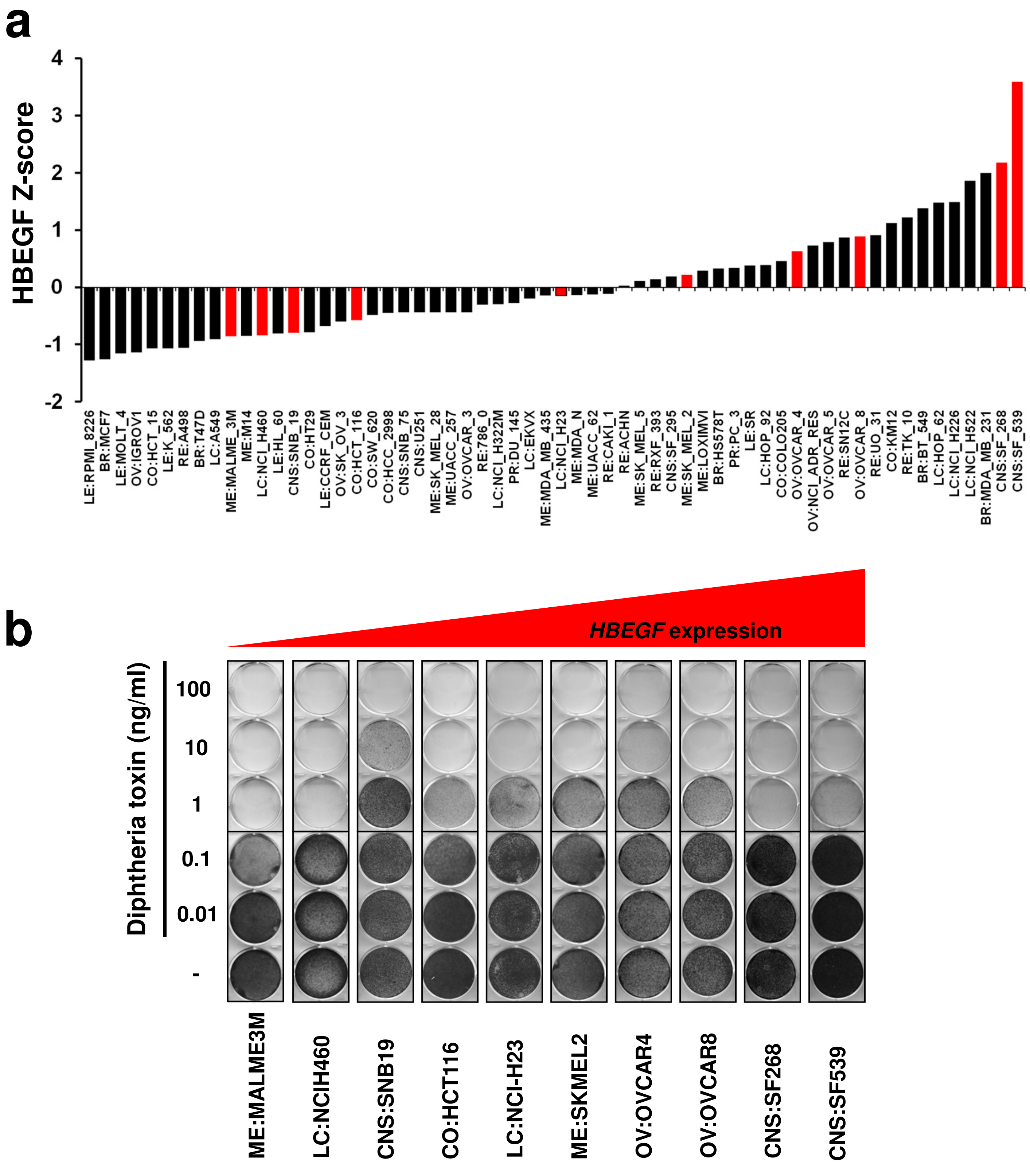

Picco et al., Supplementary Figure 2. HBEGF expression does not affect sensitivity to DT in human cancer cell lines. (**a**) Histogram representing HBEGF mRNA expression (Z-score) in the NCI60 panel of human cancer cell lines (data from the cBbioPortal: http://www.cbioportal.org). Red bars point out the ten cell lines from different tissues selected for DT treatment. (**b**) Crystal violet staining of selected cell lines, grown for one week in the presence of variable DT concentrations. Cells are ordered by increasing HBEGF expression, from left to right, as indicated by the red wedge on top.

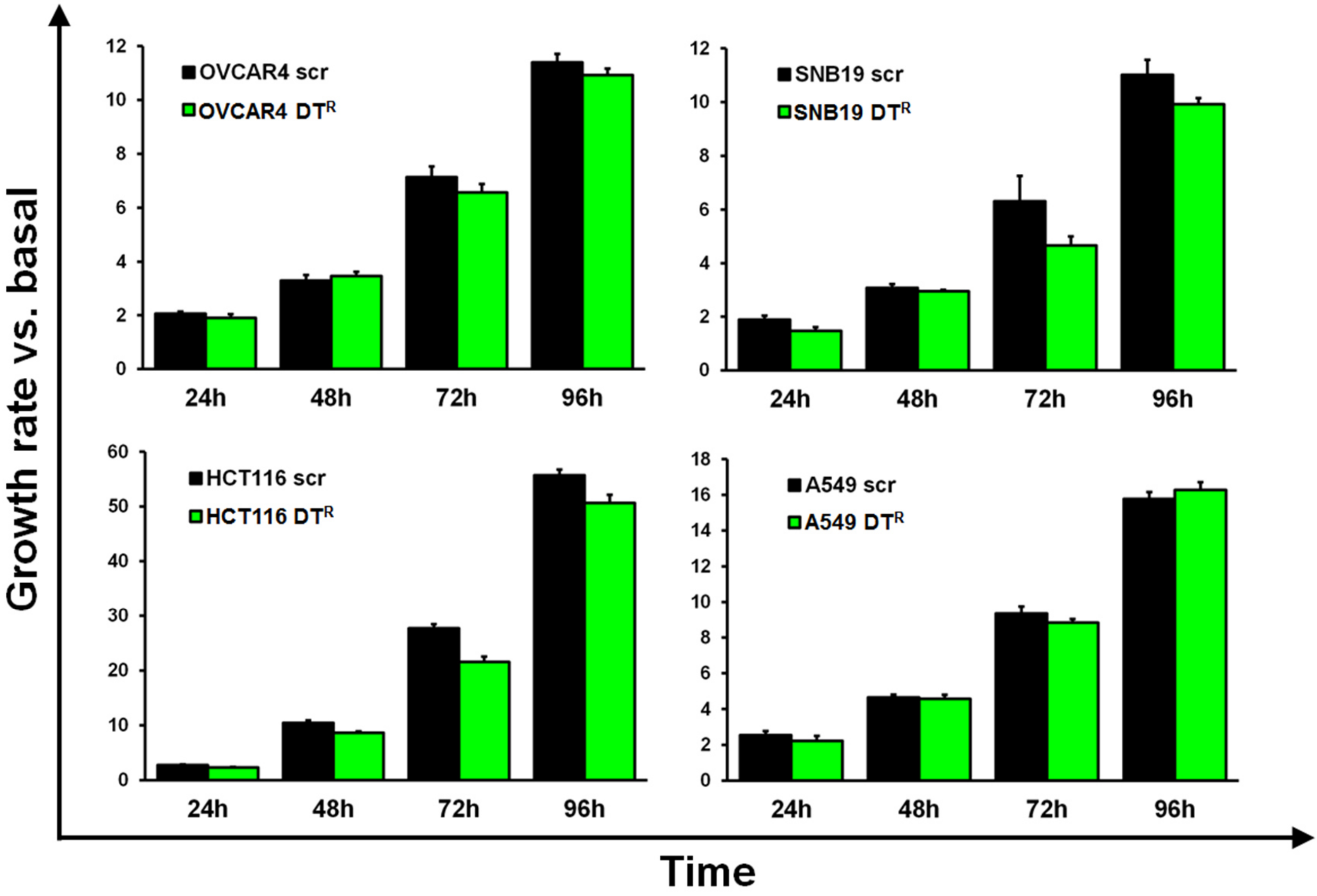

Picco et al., Supplementary Figure 3. *DPH2* silencing by DT^R does not affect basal growth rate of cancer cell lines. Histograms representing the growth of OVCAR4 (ovary), SNB19 (glioblastoma), HCT116 (colon) and A549 (lung) cancer cell lines transduced with DT^R or scramble construct. The growth rates on the y-axes were calculated by comparing ATP-based viability measurements at each time point vs. the measurements at plating.

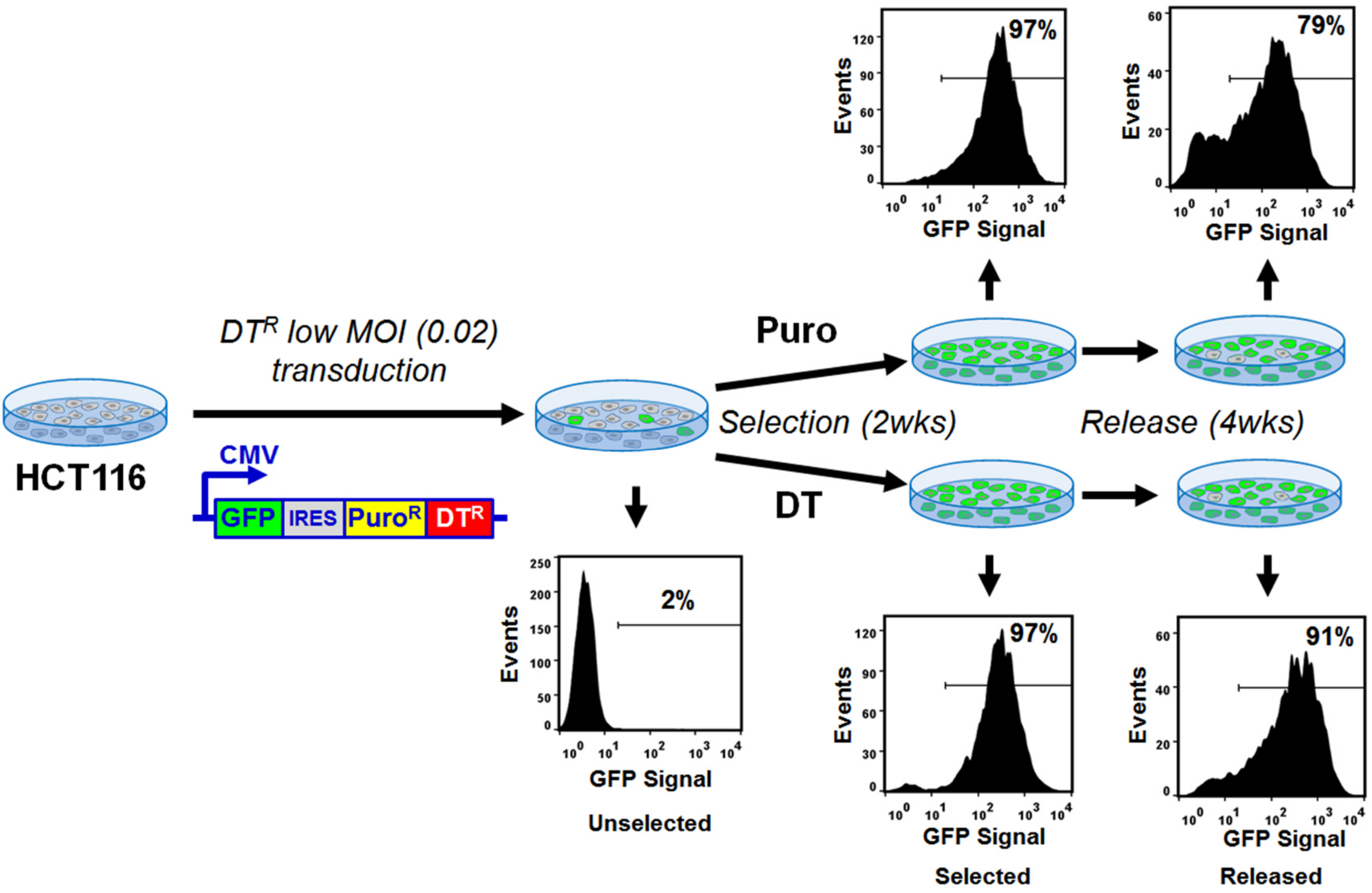

Picco et al., Supplementary Figure 4. DT^R is an efficient selectable marker *in vitro*. HCT116 cells were infected with DT^R at low MOI (~0.02), to ensure transduction of a low fraction of cells with a single copy of the vector. Three days after infection, only ~2% of the cells expressed GFP. Cells were then incubated with puromycin (2 ng/ml) or DT (10 ng/ml) for two weeks. Subsequently, selection was released for four weeks to assess stability of the GFP+ fraction. Histograms represent the distribution of GFP signal: (i) in unselected transduced cells, (ii) after puromycin/DT selection, and (iii) after release from puromycin/DT selection, as indicated.

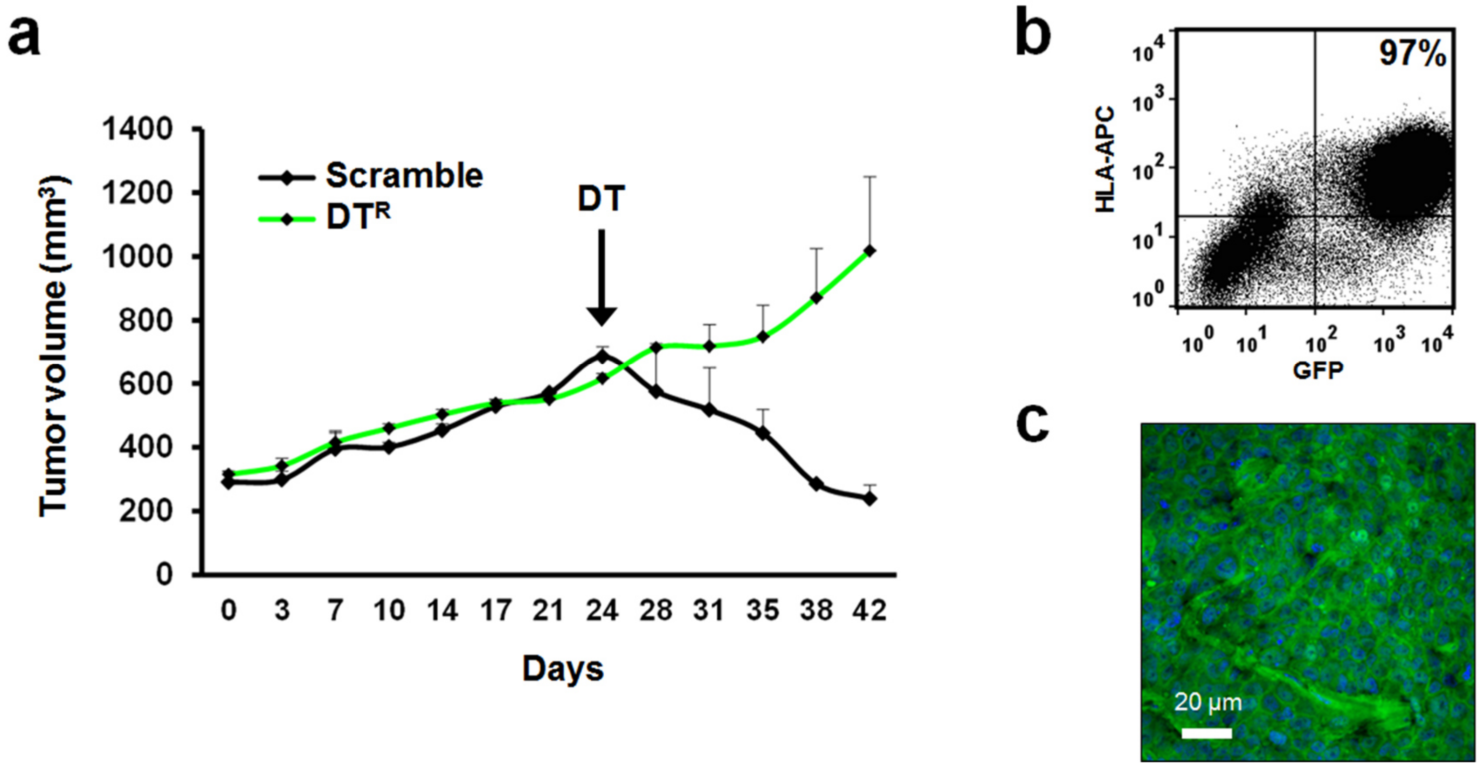

Picco et al., Supplementary Figure 5. Stability of DT resistance and GFP expression after tumor propagation. (**a**) Scramble and DT^R transduced/selected HCT116 xenografts (n = 2 per group) were re-implanted in the right flank of CD1-nude mice. When tumors reached approximately a volume of 250 mm^3, tumour growth was monitored for three weeks. At day 24, DT (5 µg/kg) was administered to mice. DT^R tumors (green line) continued growing in presence of DT, while scramble tumors (black line) rapidly reduced their volume. Flow cytometry analysis (**b**) and fluorescence micrograph (**c**) of the DT^R xenograft explanted at day 24 (before the new DT selection) revealed a very high fraction of GFP+ cells.

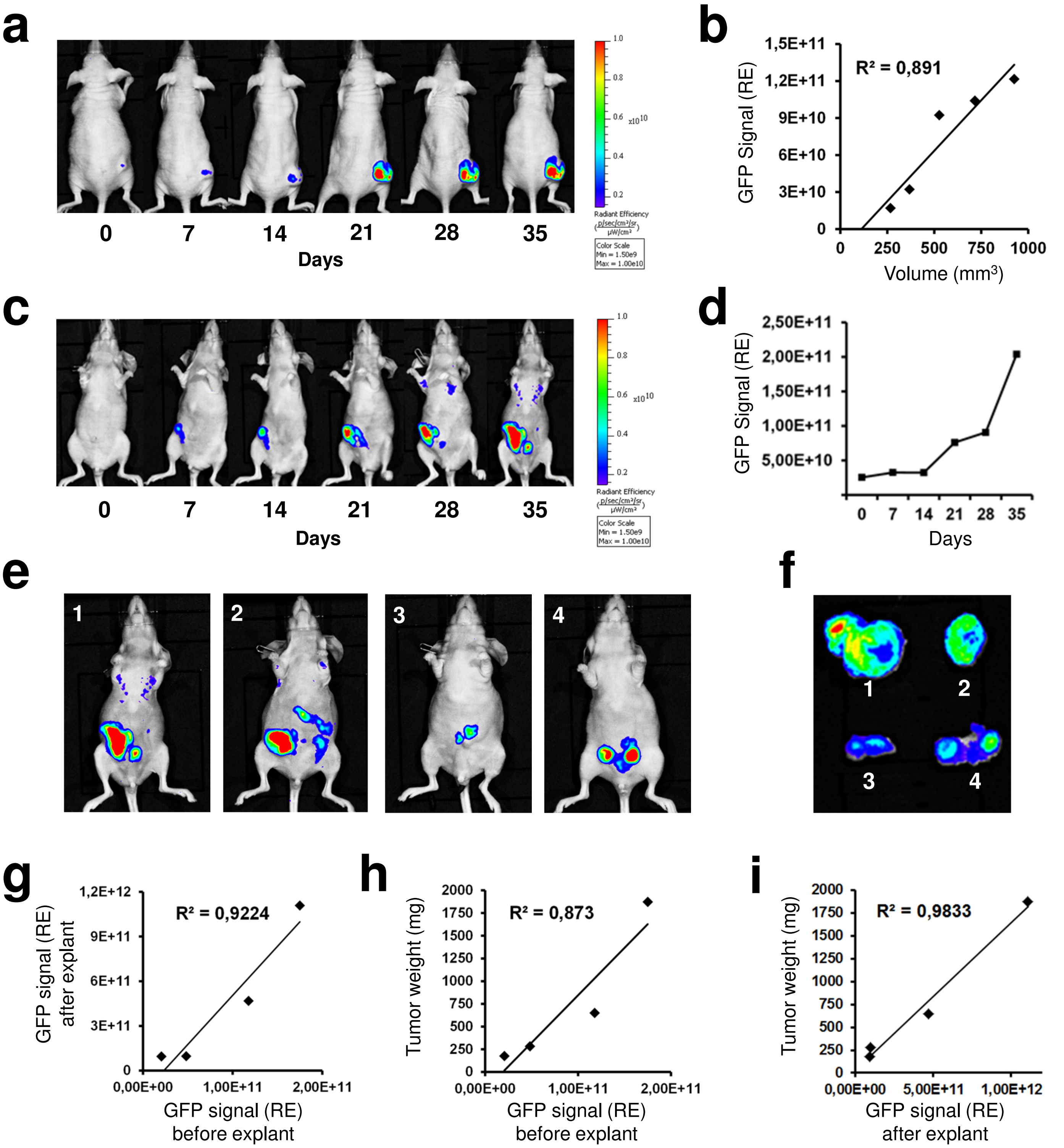

Picco et al., Supplementary Figure 6. Live imaging of DT^R-transduced HCT116 xenografts growing subcutaneously and intraperitoneally. (**a**) HCT116 xenografts transduced *in vivo* with DT^R- (GFP+) were propagated subcutaneously in CD1–nude mice. (**b**) Scatter plot representing the correlation between caliper measurements and GFP fluorescence signal (Radiant efficiency, RE) detected by live imaging in subcutaneous xenografts. (**c**) Growth of DT^R-transduced HCT116 xenografts (GFP+) propagated in the intraperitoneal (IP) cavity of CD1–nude mice. (**d**) Line chart reporting the GFP signal measured by live imaging (IVIS-Caliper) as a surrogate maker of tumor growth. (**e**) GFP signal of four DT^R IP xenografts. (**f**) GFP expression of the tumours after explant, imaged by IVIS. (**g-h**) Scatter plots comparing GFP signal before explant with GFP signal after explant or with tumor weight after explant. (**i**) Comparison of GFP signal and tumor weight, both measured after explant.

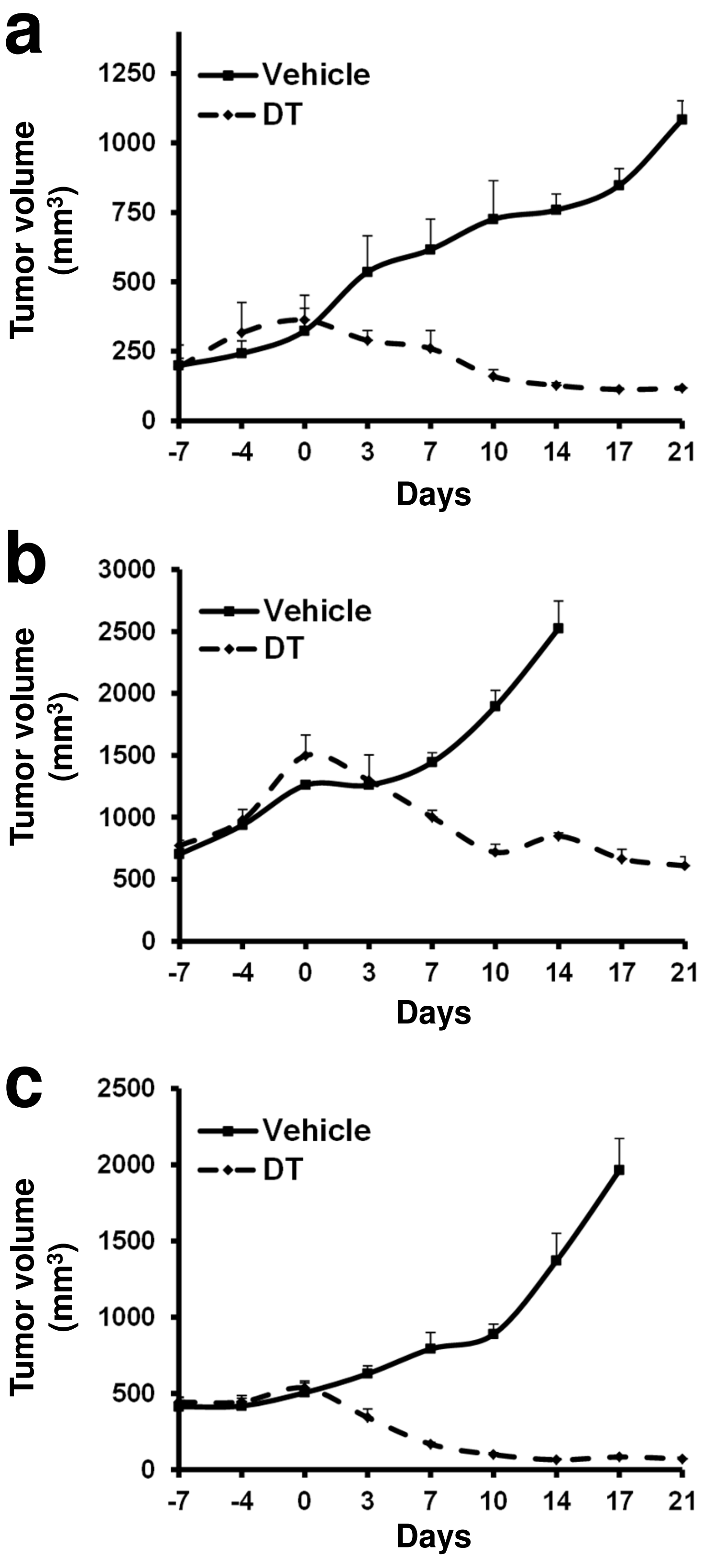

Picco et al., Supplementary Figure 7. Colorectal cancer PDXs are sensitive to DT. Patient-derived xenografts from three metastatic CRC specimens, with different KRAS/BRAF genetic backgound were grown in NOD/SCID mice. The three graphs represent tumor growth inhibition of xenografts derived from: (**a**) KRAS mutated (G13D), (**b**) BRAF mutated (V600E) or (**c**) KRAS/BRAF WT CRC tumors after administration of DT (10 µg/kg) for three weeks.

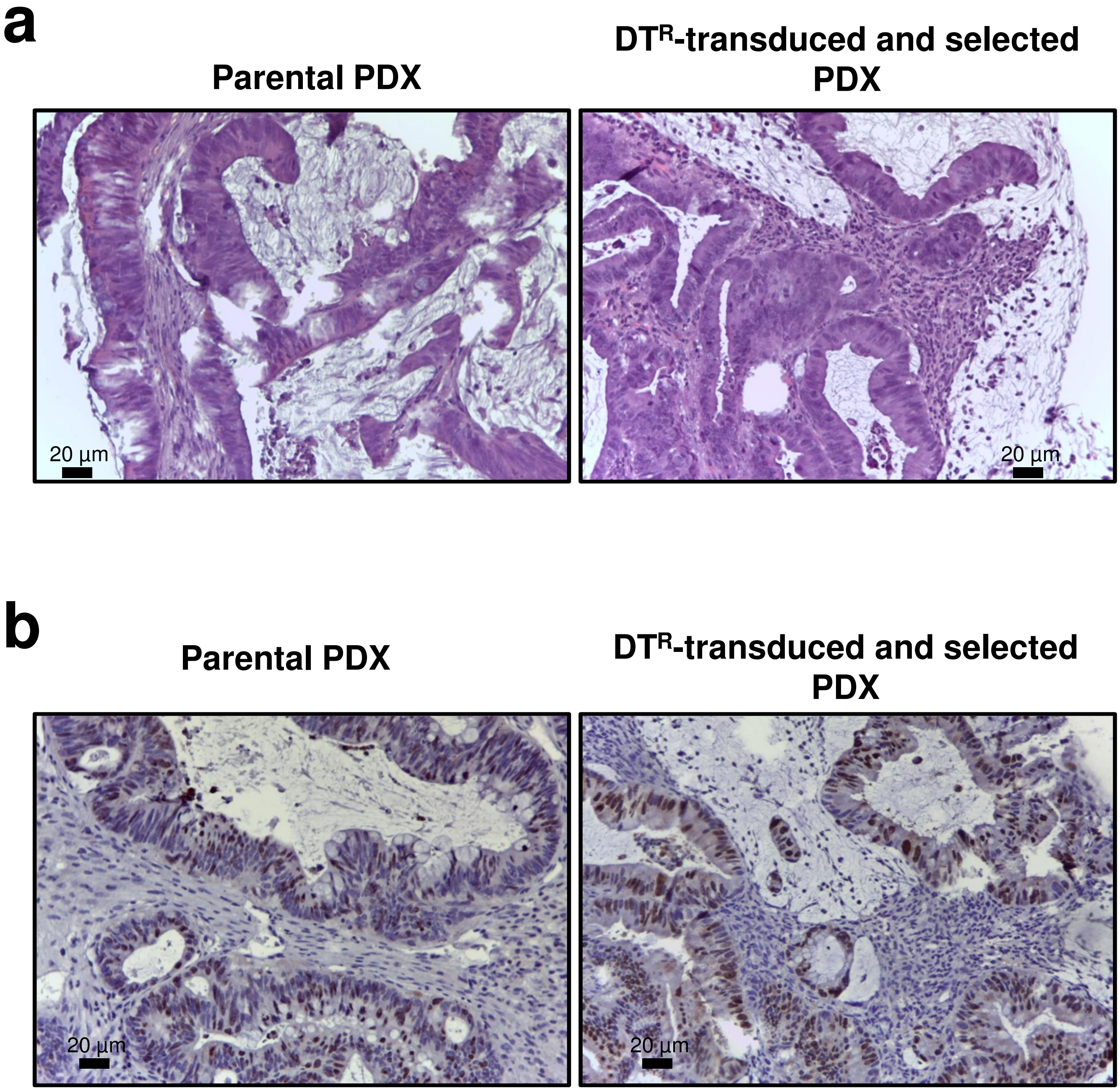

Picco et al., Supplementary Figure 8. DT^R-transduced PDXs retain histopathologic and functional characteristics of the original sample. (**a**) Haematoxylin and eosin staining of parental and DT^R-transduced PDX. (**b**) Ki67 expression in parental and DT^R-transduced PDX.

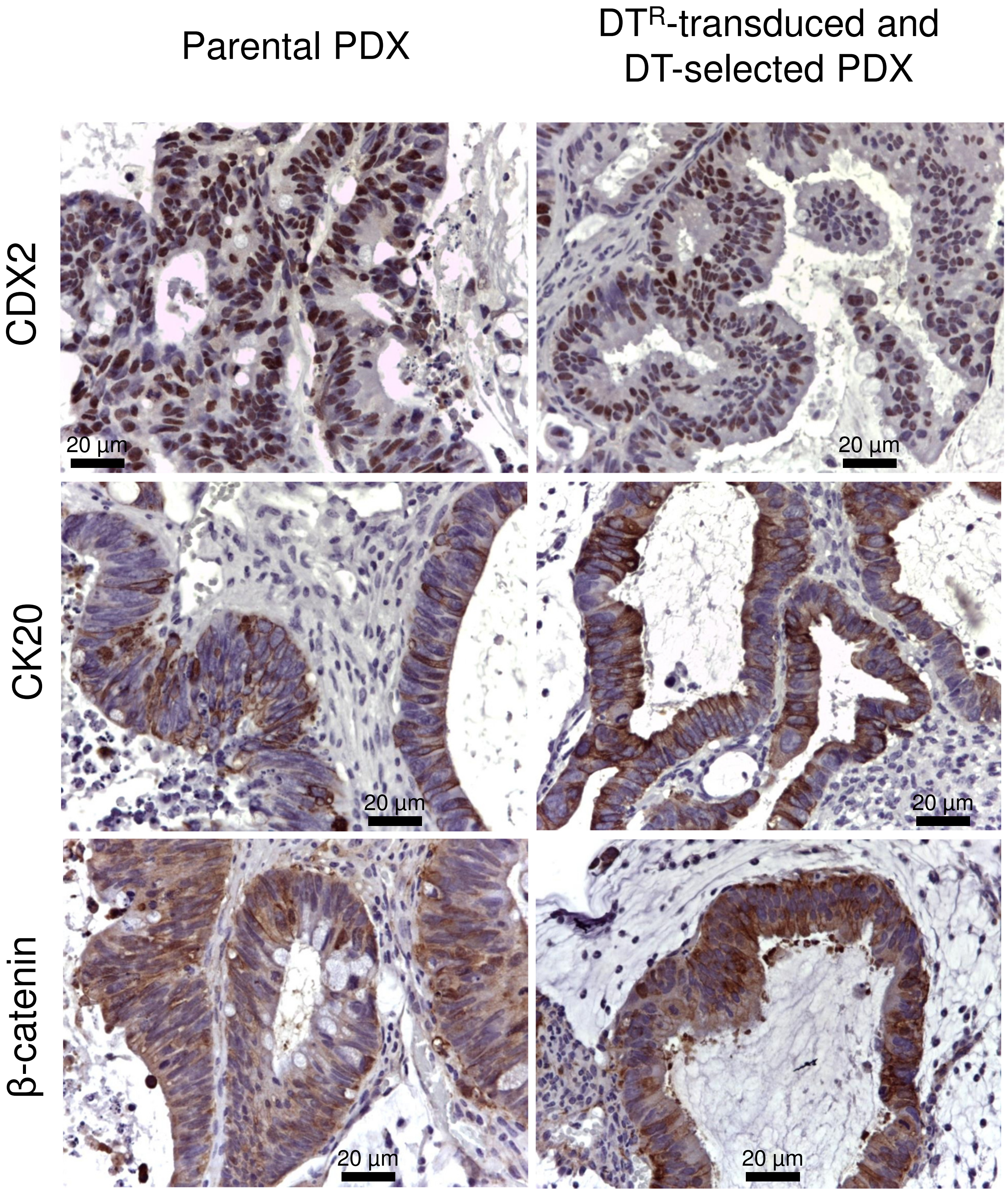

Picco et al., Supplementary Figure 9. DT^R transduction and DT selection do not significantly alter the PDX phenotype. CDX2, CK20 and β-catenin expression in a CRC PDX before (left panels) and after (right panels) DT^R transduction and DT selection.

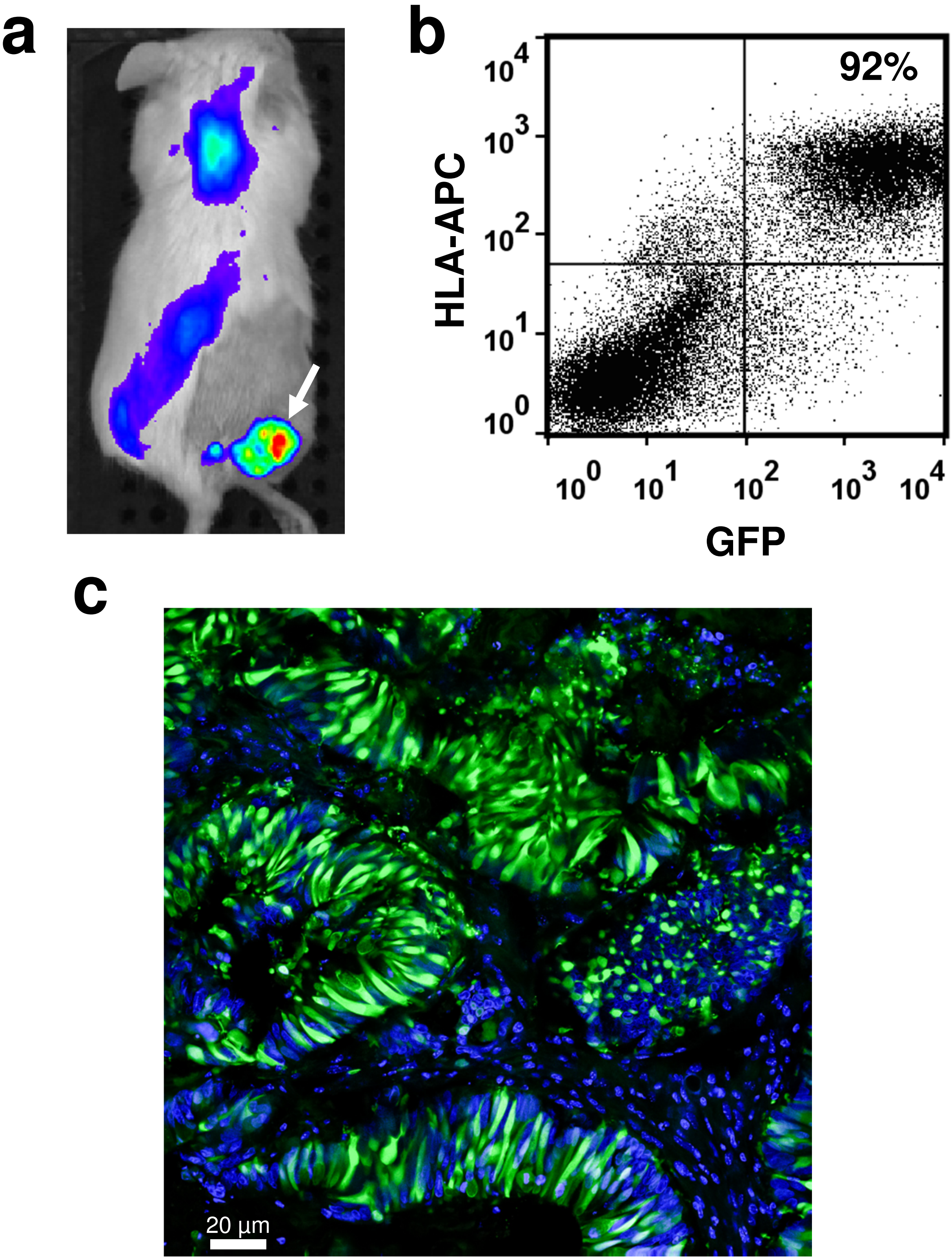

Picco et al., Supplementary Figure 10. Stability of GFP expression after tumor propagation. After DT^R transduction and DT selection, a CRC PDX was propagated for four passages in NOD-SCID mice. (**a**) Live imaging highlighting the GFP-positive tumor mass (white arrow). (**b**) Flow cytometry analysis of cells from the P4 tumor explant, displaying GFP signal on the x-axis and the HLA-APC human marker on the y-axis. (**c**) fluorescence microscopy displaying the GFP signal (green) in cancer cells vs. the nuclear DAPI signal (blue) in all cells.

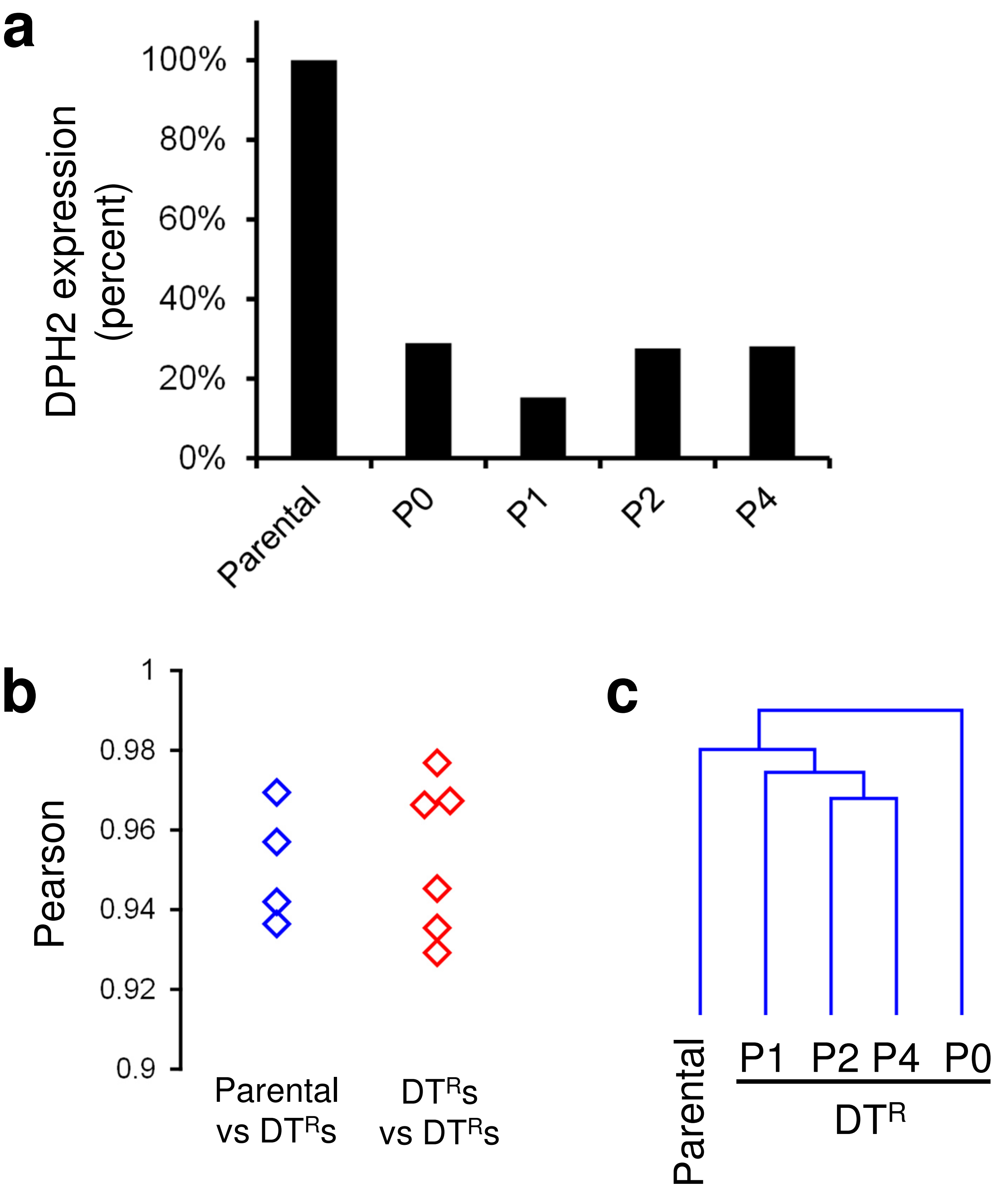

Picco et al., Supplementary Figure 11. DT^{R} trasduction and DT selection do not alter global gene expression of CRC PDX. (a) Downregulation, respect to the parental PDX, of the DPH2 transcript in all the DT^{R} derivatives passaged in the absence of DT. (b) Pearson correlation values based on global expression profiles, comparing parental PDX with DT^{R} derivatives (blue diamonds) or DT^{R} derivatives with each other (red diamonds). (c) hierarchical clustering based on global gene expression profiling of a parental PDX and its DT^{R} derivatives at different passages in mice.
